# Spatial distribution and determinants of Vitamin A supplementation non-receipt among children aged 6–35 months in Ethiopia: a multiscale geographically weighted regression analysis

**DOI:** 10.3389/fpubh.2025.1483588

**Published:** 2025-10-31

**Authors:** Altaseb Beyene Kassaw, Amare Abera Tareke, Gosa Mankelkl, Tamrat Anbesaw, Alemu Gedefie, Habtu Debash, Dawit Tesfa Tamirat, Gashaw Abebe

**Affiliations:** ^1^Department of Biomedical Science, College of Medicine and Health Science, Wollo University, Dessie, Ethiopia; ^2^Department of Psychiatry, College of Medicine and Health Science, Wollo University, Dessie, Ethiopia; ^3^Department of Medical Laboratory Science, College of Medicine and Health Science, Wollo University, Dessie, Ethiopia; ^4^Department of Health Informatics, College of Medicine and Health Science, Wollo University, Dessie, Ethiopia

**Keywords:** Vitamin A supplementation, geographical weighted regression, spatial analysis, Ethiopia, Vitamin A (carotenoids), micronutrient supplementation, child health

## Abstract

**Background:**

Vitamin A supplementation is an important public health intervention strategy to reduce childhood morbidity and mortality. However, in Ethiopia, the coverage remained low with significant regional disparities. Hence, this study aimed to explore the spatial distribution and determinants of not receiving Vitamin A supplements among children aged 6–35 months.

**Methods:**

This study utilized the 2019 Ethiopia Mini Demographic and Health Survey data conducted from March to June 2019. A total weighted sample of 2,540 children aged 6–35 months were included in the analysis. Data was managed and analyzed using STATA version 17, ArcGIS version 10.7.1, SaTScan v10.1, and MGWR version 2.2 software. A spatial autocorrelation analysis was performed to assess whether cases of failed to have Vitamin A supplements were randomly distributed or not. Hotspot analysis was performed to identify high or low prevalence, and ordinary kriging was utilized for interpolation. Furthermore, the Bernoulli-based model was used to identify the most likely clusters of not having Vitamin A supplementation by SaTScan analysis. Finally, the Geographical weighted regression and the Multiscale Geographical weighted regression analysis models were fitted to identify the spatially varying determinants of not receiving Vitamin A supplementation.

**Result:**

As the spatial analysis showed, the distribution of not having Vit-A supplements among children 6–35 months in Ethiopia was spatially varied. Clusters with the highest prevalence were identified in Sidama, Southern Nations, Nationalities, and Peoples’ Region, and some parts of Oromia. The scan statistics recognized a total of 44 primary clusters located in Sidama, Southern Nations, Nationalities, and Peoples’ Region, Southwest Ethiopia, and some parts of Oromia (Relative risk = 1.5, *p*-value < 0.001). The spatial regression analysis showed that the observed geographical variation of not having a vitamin A supplement was associated with being from an uneducated mother, being from a female household head, being from a poor/poorer household, being the first child, a female child, and a child aged 6–23 months.

**Conclusion:**

The study reveals significant geographical variation in the prevalence of not having Vitamin A supplements among children aged 6–35 months in Ethiopia. Sociodemographic factors like being from uneducated mothers, poor households, children aged 6–23, and first-born children were found to be some of the determining factors for the disparity. Hence, the need for region-specific public health interventions is highly encouraged to improve the coverage of vitamin A supplementation in Ethiopia.

## Background

Vitamin A (Vit-A) is one of the essential micronutrients required for vision, immune function, and maintenance of epithelial tissues (1). Vit-A also plays a role in cellular communication, growth regulation, and embryonic development. It has an antioxidant effect and promotes cell turnover and repair, aiding skin health (2). When the intake of Vit-A is inadequate to meet physiological requirements, it would result in a deficiency syndrome. Its severe deficiency may result in several significant health issues, including eye damage with childhood blindness, and growth retardation in children. Vitamin A deficiency also increases the severity of infections such as measles and diarrheal disease in children and slows recovery from illness (3, 4).

The World Health Organization (WHO) report states that 190 million preschoolers and 19 million pregnant mothers worldwide were exposed to Vit-A deficiency (5). It is generally acknowledged as a significant public health issue in low- and middle-income countries (4). In those nations, the deficiency is most likely to affect infants and pregnant mothers. It generally affects about one-third of under-five children, accounting for 2% of deaths in this age group (6). Despite greater efforts to reduce the incidence and impact of Vit A deficiency, the issue persists as a public health problem in developing countries, including Ethiopia, due to disparities in the food carrier, medical treatment, economy, and availability of other nutrients (7).

In countries where Vit-A deficiency is high and the under-five mortality rate exceeds 70 per 1,000 live births, the WHO recommended a semi-annual high-dose Vit-A supplement to children 6 to 59 months of age, with a targeted coverage rate of 80% (8). However, the coverage in risky areas of the world is still inadequate. According to a recent estimate based on different DHS surveys, nearly half of preschoolers in Sub-Saharan Africa did not receive the supplement. In the adequately supplemented areas, it reduced the risk of all-cause death by 12% compared to non-supplemented controls, leading to a significant reduction in child morbidity and mortality in the long run. The intervention also resulted in a 15% reduction in diarrhea incidence, a 50% decrease in measles incidence, and a 12% reduction in diarrhea-related mortality (6, 9).

Supplementation of Vit-A for children is often integrated with routine immunization programs and vaccination campaigns, providing additional benefits through enriched breast milk delivery. In Ethiopia, the government has carried out widespread Vit-A supplementation campaigns in collaboration with foreign organizations. A regular delivery of high-dose Vit-A oral supplements has been implemented in preschool children through the campaign-based approach. The program has been implemented since 2004, resulting in an improvement in the coverage (10). However, the supplementation coverage has continually become low (11–14) since the campaign-based delivery was replaced by a routine delivery system in 2010 (15). According to the DHS report, the national coverage of receiving the supplement for eligible children was 56% in 2011 and 45% in 2016 (16, 17). The proportion was even very low in certain areas of the country.

Even though national efforts have been undertaken to improve the coverage, a high proportion of eligible children in Ethiopia have not received this essential supplement, which increases the risk of morbidity and mortality. Hence, exploring the spatial distribution and its important local determinants is credible. By employing a spatial analysis model, this study would offer the identification of a high-risk area and regionally varying socioeconomic/demographic factors that could affect the likelihood of not obtaining a Vit-A supplement. Identifying the specific locations and local determinants is used for designing targeted and area-specific interventional strategies that may tackle the distinct barriers encountered by different regions.

## Methods and materials

### Data source and study area

This research was based on the 2019 Ethiopia Mini Demographic and Health Survey (EMDHS), which was carried out between March and June of 2019. The Federal Ministry of Health (FMoH), the Central Statistical Agency (CSA), and the Ethiopian Public Health Institute (EPHI) collaborated to conduct the program. The Ethiopia Demographic and Health Survey is nationally representative conducted every 5 years in Ethiopia. Ethiopia has 9 regional states (Afar, Amhara, Benishangul-Gumuz, Gambela, Harari, Oromia, Somali, Southern Nations, Nationalities, and People’s Region (SNNP), and Tigray) and two Administrative Cities (Addis Ababa and Dire-Dawa). The data were downloaded from the DHS program website, https://dhsprogram.com/data/dataset, after permission had been obtained. Only this study used the accessed data, and no third party was given access without first registering.

### Sampling procedure

The sampling frame for the 2019 EMDHS was based on the 2019 Ethiopia Population and Housing Census (EPHC) conducted by the Central Statistical Agency (CSA) of Ethiopia (18). The census frame included a complete list of 149,093 enumeration areas (EAs), which contained information on EA location, type of residence (urban or rural), and the estimated number of residential households. Administratively, Ethiopia is divided into nine regions and two administrative cities, and the sample was designed to provide estimates at the national level, for urban and rural areas separately, and for each region and administrative city.

**Figure 1:**
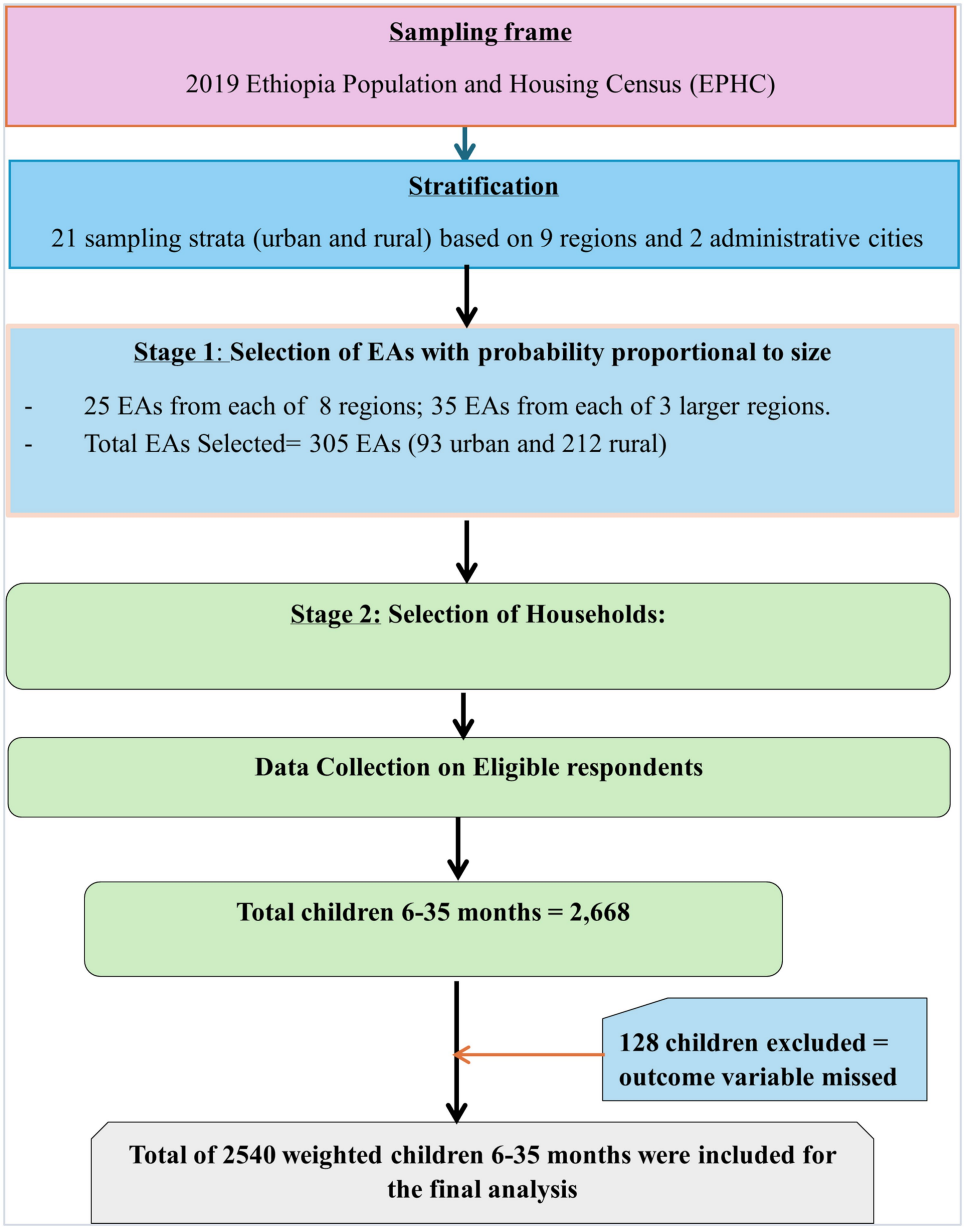
Sampling producer of non-receipt of Vit-A supplement among children aged 6–35 months in Ethiopia, EMDHS 2019.

The survey utilized a two-step stratified cluster sampling procedure. In the first stage, each of the nine regions and two administrative cities was divided into urban and rural areas, yielding 21 sampling strata. Within each stratum, enumeration areas (EAs) were selected independently using probability proportional to size, with implicit stratification and proportional allocation applied at lower administrative levels by sorting the sampling frame within each stratum. To ensure comparable survey precision, 25 EAs were selected from each of the eight regions, while 35 EAs were selected from each of the three larger regions, Amhara, Oromia, and SNNPR, resulting in a total of 305 EAs (93 urban and 212 rural EAs, with probability corresponding to EA size). In the second stage, after a complete household listing in each EA, a fixed number of 30 households per cluster were selected using systematic sampling. Interviews were available for all mothers or caregivers aged 15 to 49 who were either long-term residents of the chosen homes or visitors who spent the night before the survey (18) (Figure 1).

### Study population and data extraction

The source population for this study was children of the age range 6–35 months old who lived with the respondent (women aged 15–49 years). Different datasets, including those on males, women, children, births, and households, were included in the survey. We used the Kids Record dataset (KR file). Finally, a total of 2,540 weighted children aged 6–35 months were taken into account for the analysis.

### Study variables

All variables in this study were selected from peer-reviewed literature and the 2019 EMDHS. These variables were coded and categorized based on the Demographic and Health Survey (DHS-7) recode manual.

The outcome variable was not receiving Vit A supplement. This outcome data was collected from mothers’ direct verbal reports, whether their children had taken vitamin A capsules or not.

The predictor variables were child age, child sex, child twin status, parity, place of delivery, ANC visit, PNC visit, residence, maternal age, current marital status, maternal educational level, religion, household wealth index status, sex of household head, and media exposure.

### Data management and analysis

Before any analysis, the data were weighted to ensure the representativeness of the sample and to obtain a reliable statistical estimate. The STATA version 17 software was used for descriptive analysis and management. Descriptive statistics, including percentages and frequencies, were applied to describe the background of the study participants. ArcGIS version 10.7.1, SaTScan v10.1 software, and MGWR version 2.2 were used for spatial data analysis.

### Spatial autocorrelation analysis

Global Moran’s I measure was used to verify whether non-receipt of Vit- A supplement was clustered, dispersed, or randomly distributed in Ethiopia. Global Moran’s I calculates Moran’s I Index value, Z score & *p* value. Moran’s I index close to −1 means cases not receiving Vit-A supplement were dispersed, whereas close to 1 indicates that cases are clustered and not receiving Vit-A supplement distributed randomly if the I value was zero. Statistically significant Z-score and *p* value≤0.05 indicated the existence of clustering. Statistically non-significant Moran’s I value (if *p* value>0.05) indicates cases of not receiving Vit-A supplements were randomly distributed throughout the country.

### Hotspot analysis

Hotspot analysis was performed by calculating the GI* statistic (Getis-OrdGi* statistic for each area and statistical outputs with high GI* indicate “hotspot” areas, while low GI* indicates “cold spot” areas). The GI* statistic is a z-score. A high Z score and a small *p*-value for a feature suggest a significant hotspot. A significant cold spot area was indicated by a small p-value and a low negative z-score. The intensity of the clustering increases with a greater (or lower) z score. A z-score near zero means no spatial clustering. Thus, using this methodology, statistically significant regional clusters of high values (hotspots) and low values (cold spots) of children aged 6–35 months not getting Vit-A supplements were identified.

### Spatial scan statistical analysis

We performed a Bernoulli-based spatial scan analysis to identify the geographic locations of statistically significant clusters for non-receipt of Vit-A supplement among children 6–35 months using SaTScan™ software version 10.1.3. The spatial scan statistic uses a circular scanning window that includes statistically significant spatial clusters for Vit-A supplementation non-receipt. Children aged 6–35 months old who had not received Vit-A supplements were included in the model as cases, and children who received included in the control group. A binary variable with values of 0 and 1 was employed. The circle with the highest likelihood was the primary cluster (most likely cluster). The remaining significant clusters are considered secondary clusters, which are generated by removing the primary clusters automatically in the SaTScan software and by selecting no geographic overlap during analysis, and we ordered them according to their likelihood ratio test statistics. The maximum cluster size was set at 50% of the population at risk (19). For each cluster, the relative risk (RR), population, location radius, cases, and log-likelihood ratio (LLR) test statistics with *p*-value were presented.

### Spatial interpolation

Spatial interpolation is a process in which a map surface is created by estimating the values at unsampled points based on known values of the surrounding sampled points (20). Among the various methods of interpolation, the Ordinary Kriging technique was used in this study to predict the likelihood of not receiving Vit- A supplements among children aged 6–35 in unobserved areas.

### Spatial regression

To identify factors affecting the observed regional variations in not receiving Vit-A supplements, we employed spatial regression analysis’s global and local modeling approaches.

### Global spatial regression

The ordinary least squares (OLS) regression analysis is a global model that was applied as a preliminary test of the correlation between the dependent and independent variables It assumes a stationary and constant relationship over space, which implies that the relationships do not vary over space [i.e., it predicts only one coefficient per independent variable over the entire research area (21)]. Then, model performance and assumptions were assessed using R-square, VIF, autocorrelation of residuals, anticipated sign for coefficients, and Koenker and Jarque-Bera test statistics.

The OLS model equation is as follows:

Y = β0 + β1X1 + β2X2……. βnXn + *ε*, where β0 intercept (coefficient without explanatory variables), β1 to βn are coefficients for X1 to Xn explanatory variables and ε is the residual.

### The local spatial regression model

A local form of regression called geographically weighted regression (GWR) is used to model relationships that vary in space. GWR is used when the Koenker statistics are significant, suggesting that the associations between the covariates and the outcome vary from location to location. It assumes that the explanatory factors and the outcome have nonstationary spatial correlations, with coefficients changing at about the same pace throughout the research region. Moreover, it only employs a single constant bandwidth (22).

*The model equation for GWR is as follows:*

𝑦𝑖 = 𝛽0𝑖 + 𝛽1𝑖𝑥1𝑖 + 𝛽2𝑖𝑥2𝑖+ … + 𝛽𝑘𝑖𝑥𝑘𝑖 + 𝜀 𝑖, where 𝑦𝑖 is the value of the dependent variable at location 𝑖, 𝛽0𝑖 is the local intercept, 𝑥𝑘𝑖 is the observation of the 𝑘^th^ explanatory variable at location 𝑖, 𝛽𝑘𝑖 is the 𝑘^th^ coefficient estimate calibrated for location i, and 𝜀𝑖 is the random error term for 𝑖 = {1, 2….n (22).

We also applied the Multiscale Georgical Weighted Regression (MGWR) model to calibrate the parameter estimates of GWR using MGWR version 2.2. The MGWR is an extension of GWR that allows different processes to vary over space at different spatial scales. Rather than using a single, constant bandwidth across the whole study region, this model uses multiple bandwidths and allows the relation between the dependent and independent variables to vary spatially across many spatial scales. The adaptive bi-square kernels were utilized for geographical weighting to estimate local parameter estimates. The golden section search method was applied to determine the optimal bandwidth size based on the AICc.

*The MGWR model is specified as follows:*

$\begin{array}{l} \mathrm{yi}=\mathrm{bw}0\;(\beta 0i)+\mathrm{bw}1\;(\beta 1\mathrm{ix}1i)+\mathrm{bw}2\;(\beta 2\mathrm{ix}2i)+\ldots \\ +\mathrm{bwk}(\text{βkixki})+\varepsilon \;i \end{array}$

where 𝑦𝑖 is the value of the predictor variable at location 𝑖, 𝛽0𝑖 is the local intercept, 𝑥𝑘𝑖 is the observation of the 𝑘^th^ explanatory variable at location 𝑖, 𝛽𝑘𝑖 is the 𝑘^th^ coefficient estimate calibrated for location i, and 𝜀𝑖 is the random error term for 𝑖 = (1, 2, …n). Except for the label bw, which indicates the various bandwidths for each variable, the parameters are the same with GWR (23). Model performance was compared using corrected Akaike’s Information Criterion (AICc) and Adjusted R-squared (24).

Ethical consideration.

The data were downloaded from the DHS program website, https://dhsprogram.com/data/dataset, after permission had been obtained. The title and concept note of the research proposal were sent via the DHS website to register and obtain authorization to access the data set. Then, permission to utilize the EMDHS data was obtained through an authorization letter from ICF International. Residential addresses or names were not included in the dataset.

## Result

### Background characteristics of the study participants

This study included a weighted sample of 2,540 children aged 6–35 months, with a mean age of 20.13 ± 8.36. About 59.5% of the children were in the age group 6–23 months, and of the total participants, 1,298 (51.1%) were male. Most of the participants, 1863 (73.3%), were residing in rural areas. More than half of the children’s mothers, 1362 (53.6%), were in the age group 25–34, and nearly half of the mothers, 1,250 (49.2%), were not attending education. A detailed description is presented in Table 1.

**Table 1:** Background characteristics of the study participants, Vit-A supplement non-receipt among children aged 6–35 months in Ethiopia, EMDHS 2019.

Variables		Weighted frequency (*N*)	Percentage (%)
Child age (in months)	6–23	1,513	59.5
	24–35	1,028	40.5
Child Sex	Female	1,242	48.9
	Male	129	51.1
Child twin status	Single	2,492	98.1
	Multiple	49	1.9
Residence	Urban	678	26.7
	Rural	1863	73.3
Maternal age	15–24	687	27.0
	25–34	1,362	53.6
	35–49	491	19.4
Current marital status	Married/living with a partner	2,424	95.4
	Other	117	4.6
Place of delivery	Home delivery	1,224	48.2
	Institutional delivery	1,317	51.8
Parity	1–2	1,053	41.4
	3–5	919	36.2
	> = 6	569	22.4
Number of ANC visits	No ANC visit	560	22.0
	1–3 visit	725	28.5
	> = 4 visit	992	39.0
PNC visit	No	1937	76.2
	Yes	330	13.0
Maternal educational level	No education	1,250	49.2
	Primary	967	38.1
	Secondary	211	8.3
	Higher	113	4.4
Religion	Orthodox	877	34.5
	Catholic	11	0.4
	Protestant	700	27.6
	Muslim	912	35.9
	Traditional/Other	40	1.6
Household wealth index status	Poorer/Poorest	1,087	42.8
	Middle	497	19.6
	Richer/Richest	956	37.6
Sex of household head	Male	2,205	86.8
	Female	336	13.2
Household media exposure	No Media Exposure	1,645	64.8
	Have Media Exposure	895	35.2
Region	Tigray	167	6.6
	Afar	39	1.5
	Amhara	518	20.4
	Oromia	1,001	39.4
	Somali	167	6.6
	Benishangul	31	1.2
	SNNPR	503	19.8
	Gambela	11	0.4
	Harari	8	0.3
	Addis Adaba	83	3.2
	Dire Dawa	14	0.6

### Prevalence of non-receipt of Vit-A supplement

About 53.2% (95% CI: 51.0, 55.1%) of children aged 6–35 months had not received vitamin A supplements in the 6 months before the survey. Most of cases, 1,017 (75.3%), were from rural residents (Table 2).

**Table 2:** Prevalence of Vit-A supplement non-receipt among children aged 6–35 months in Ethiopia, EMDHS 2019.

Variables		Vitamin A supplementation - Weighted frequency (*N*), %	
		No (*N* (%))	Yes (*N* (%))
Child age (in months)	6–23	841 (55.6%)	672(44.4%)
	24–35	510 (49.6%)	518 (50.4%)
Child Sex	Male	693 (53.3%)	606 (46.7%)
	Female	658 (53.0%)	584 (47.0%)
Residence	Urban	334 (49.3%)	343 (50.7%)
	Rural	1,017 (54.6%)	846 (45.4%)
Region	Tigray	62 (37.1%)	105 (62.9%)
	Afar	23 (59.0%)	16 (41.0%)
	Amhara	204 (39.4%)	314 (60.6%)
	Oromia	550 (54.9%)	451 (45.1%)
	Somali	128 (76.6%)	39 (23.4%)
	Benishangul	11 (36.7%)	19 (63.3%)
	SNNPR	314 (62.4%)	189 (37.6%)
	Gambela	4 (36.4%)	7 (63.6%)
	Harari	4 (50.0%)	4 (50.0%)
	Addis Adaba	45 (54.9%)	37 (45.1%)
	Dire Dawa	6 (40.0%)	9 (60.0%)
		1,351 (53.2%)	1,189(46.8%)

### Spatial distribution of non-receipt of vitamin A supplement

**Figure 2:**
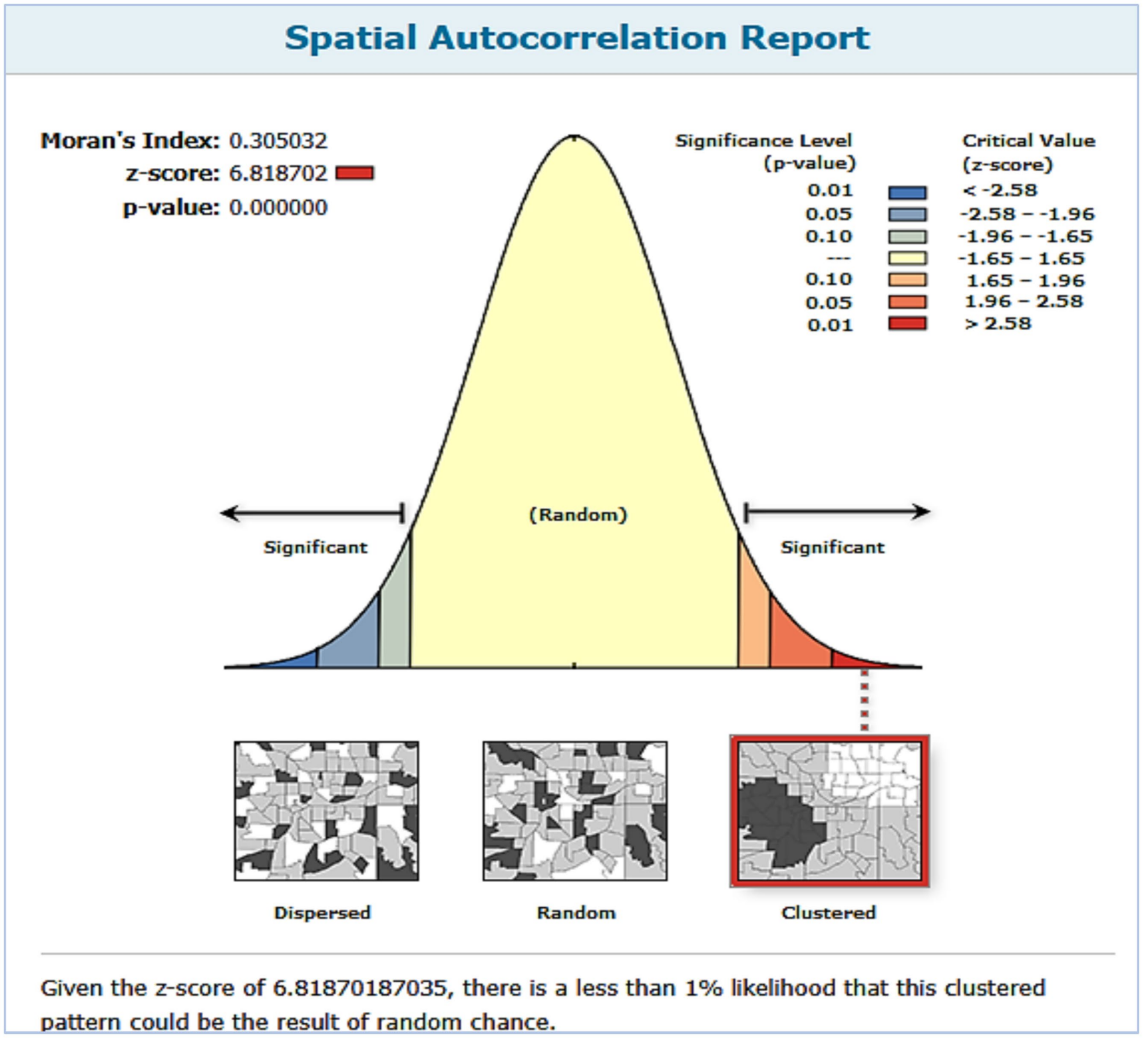
Spatial autocorrelation analysis of non-receipt of Vit-A supplement among children aged 6–35 months in Ethiopia, EMDHS 2019.

**Figure 3:**
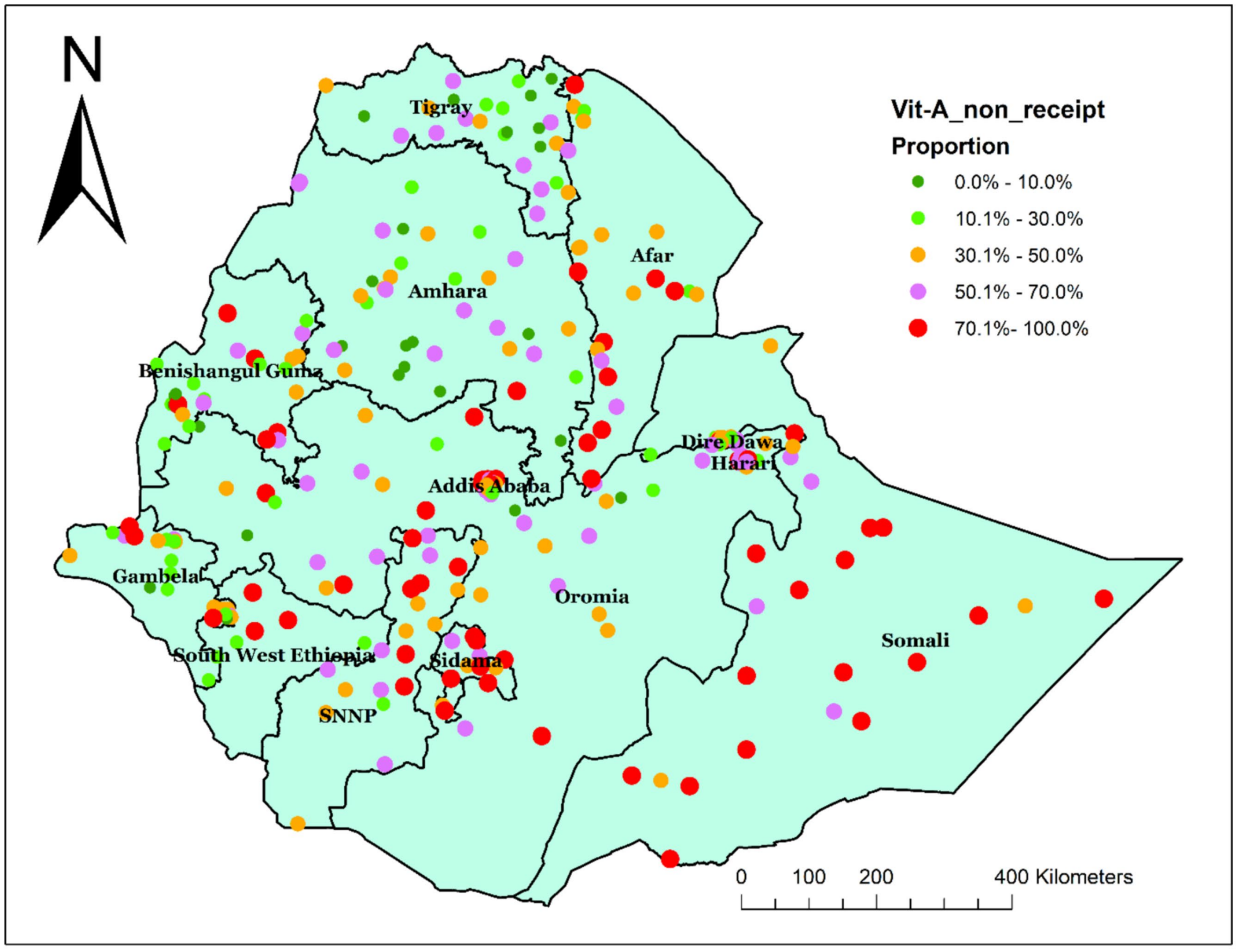
Spatial distribution of non-receipt of Vit-A supplement among children aged 6–35 months in Ethiopia, EMDHS 2019.

In Ethiopia, the spatial distribution of Vit A non-receipt among children 6–35 months of age was nonrandom. Across the country, it was spatially clustered with Global Moran’s I value of 0.305032 (*p* < 0.001) and a z-score of 6.818703, which indicated that there is less than 1% probability of the cluster due to the result of chance (Figure 2). Incremental spatial autocorrelation was performed to identify the maximum clustering. At a starting distance of 155207.63 meters, a total of 20 distance bands were detected, and the first maximum peak (clustering) was noted at 275416.39 meters (z value: 22.045291) (Supplementary file 1). Figure 3 displays the geographical distribution of non-receipt of Vit A supplements in Ethiopia among children 6–35 months of age.

### Hot spot analysis of non-receipt of Vit-A supplement

**Figure 4:**
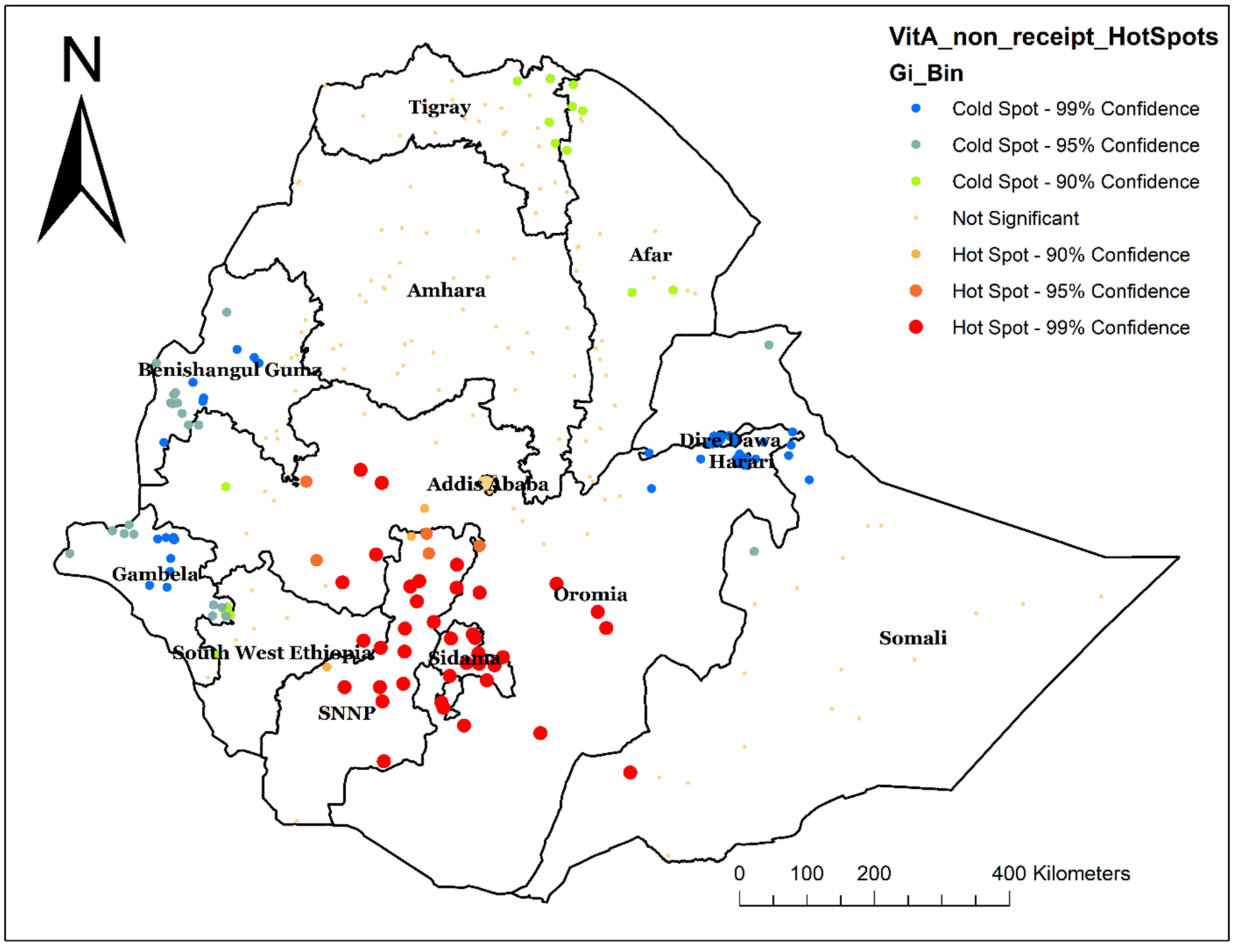
Hot spot analysis of not receiving Vit-A supplementation among children aged 6–35 months in Ethiopia, EMDHS 2019.

The hot spot analysis was performed to identify high-risk areas for not receiving Vit-A supplements. The red color, which indicates the significant hotspot areas (high prevalence of non-receipt of Vit-A supplement) was found in Sidama, SNNPR, and some parts of Oromia. Whereas the blue color indicates the cold spot area (low prevalence of non-receipt of Vit-A Supplement), which was found in Dire Dawa, Harari, Benshangul Gumz, and Gambella Regions (Figure 4).

### Spatial interpolation

**Figure 5:**
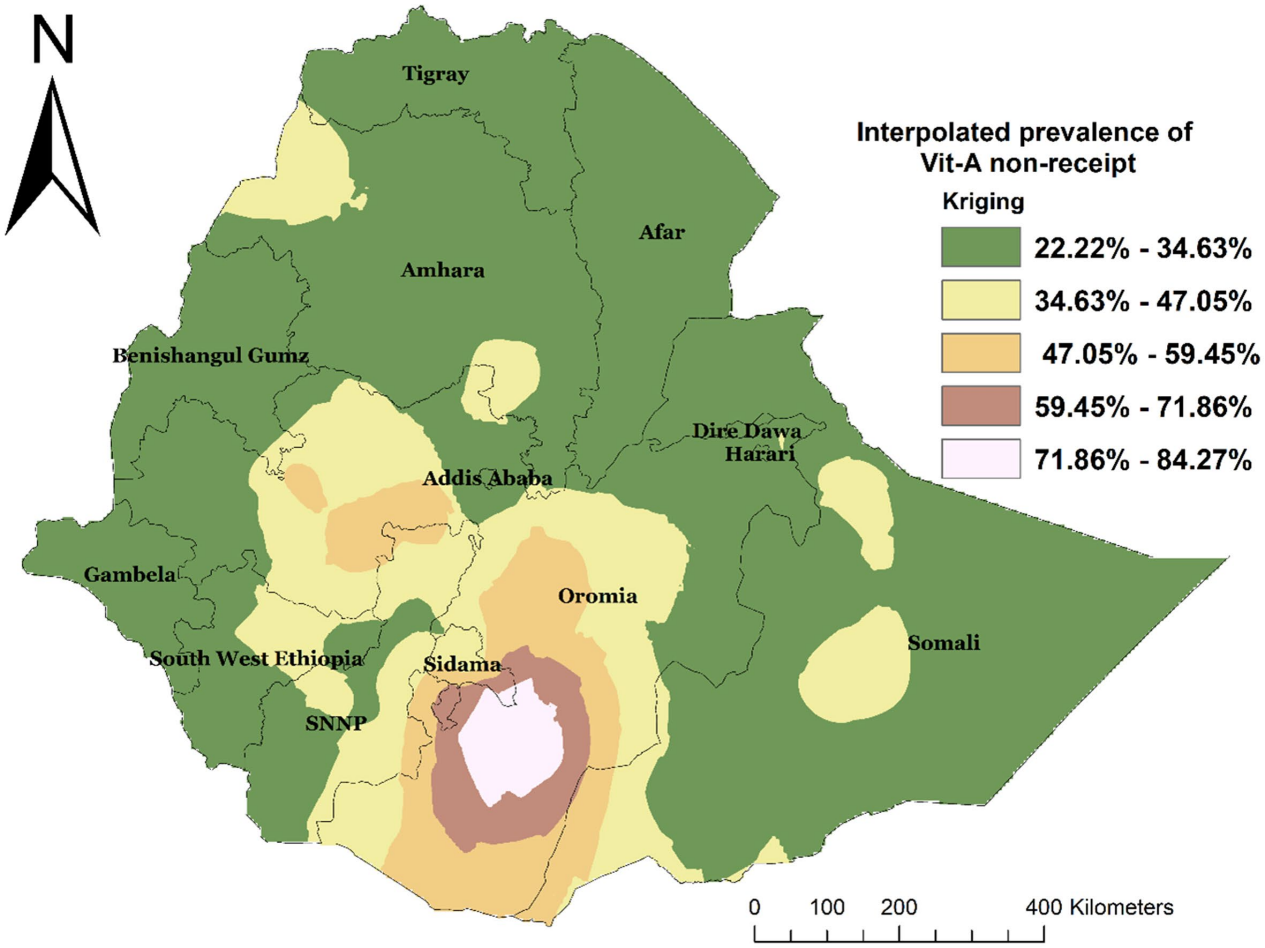
Interpolated spatial distribution of not receiving Vit-A among children 6–35 months in Ethiopia, EMDHS 2019.

Spatial interpolation was carried out to estimate the expected area of non-receipt of Vit-A supplements at unsampled sites within an area covered by the current observation. Based on the EMDHS 2019 sampled data, spatial interpolation predicted the highest rates of not receiving Vit-A supplement in children aged 6–35 months in Oromia and some parts of Sidama. In contrast, the lowest prevalence was predicted in most of the Amhara, Somali, Gambella, Afar, Harer, Diredawa, Benshangul Gumz, Tigray, and some parts of the Southwest Ethiopia Region (Figure 5).

### Spatial sat scan analysis

**Figure 6:**
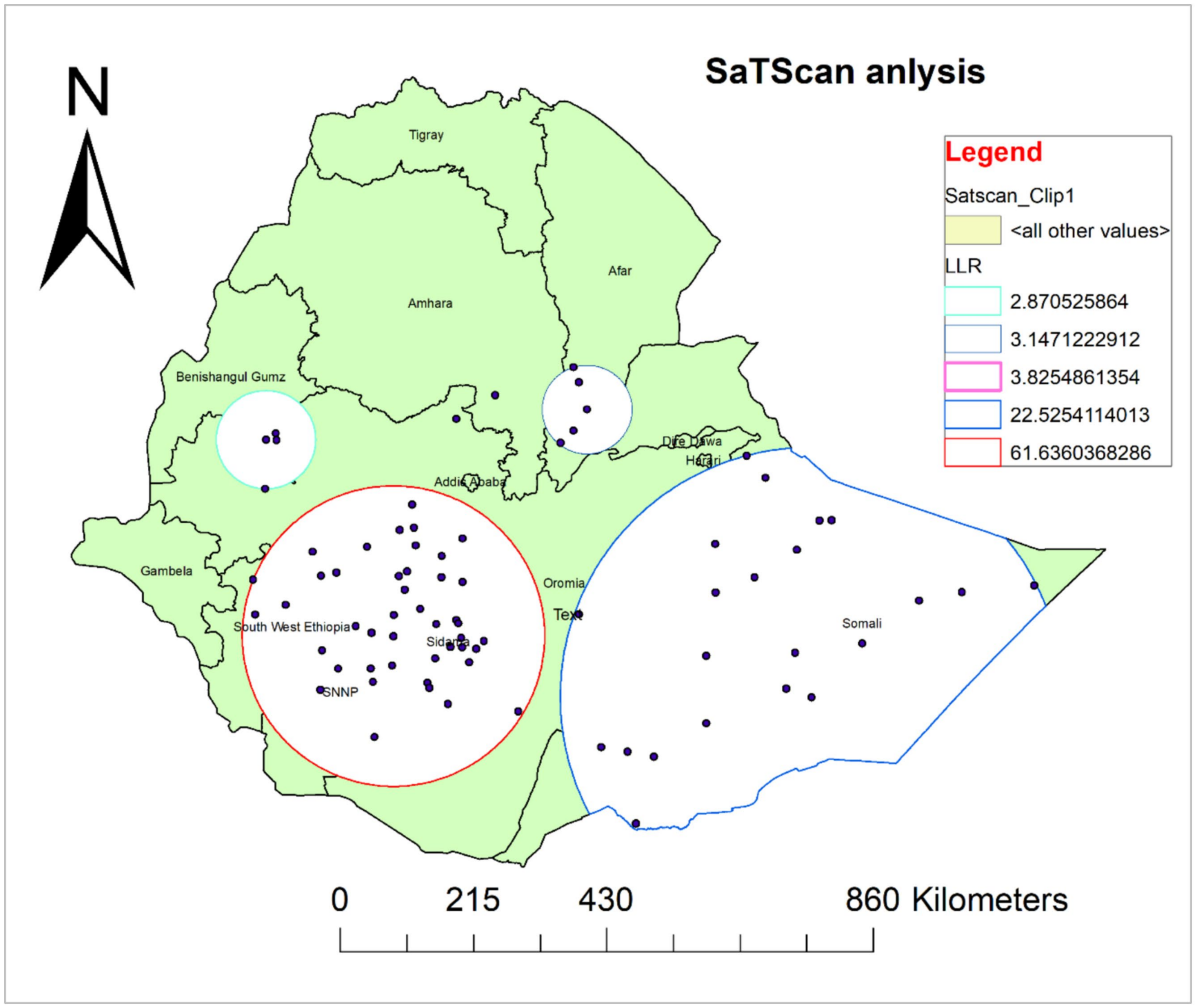
Spatial scan analysis of not receiving Vit-A supplement among children aged 6–35 months in Ethiopia, EMDHS 2019.

A total of 66 significant clusters were identified, of which 44 were most likely (primary) clusters and 22 were secondary clusters. The spatial window for the primary clusters was located in Sidama, SNNP, Southwest Ethiopia, and some parts of Oromia. It was found at (6.745024 N, 37.662745 E)/(241.91 km) radius. In the primary cluster, 610 (68.1%) of children 6–35 months have not received Vit-A supplement, and those children in this cluster were 1.5 times more likely not to receive Vit-A supplement compared with those outside this window (RR = 1.5, LLR = 61.36; *p*-value<0.001). The second most likely SaTScan cluster covered mainly the Somali region and the eastern and southeastern Oromia region, which was located at (5.856584 N, 43.726016 E)/(402.89 km) radius. Clusters in the second SaTScan window were also 1.51 times more likely not to receive Vit-A compared with those outside this window (RR = 1.51, LLR = 22.53; *p*-value<0.001) (Supplementary file 2) and (Figure 6).

Spatial regression analysis for factors affecting spatial variations of non-receipt of Vit-A supplement among children aged 6–35 months.

### Results from ordinary least squares (OLS)

The OLS model was employed to investigate the assumptions of spatial regression and the coefficients of selected predictor variables (Table 3). The model yields statistically significant results as evidenced by the Joint F-statistic and Wald Statistic (*p*-value <0.001). The model explained 85.33% of the variation in not receiving Vit-A supplements among children aged 6–35 months (Adjusted R^2^ = 0.853305) with Akaike’s Information Criterion (AICc) 1505.93. Furthermore, the Koenker (BP) statistics were also found to be statistically significant, indicating that the relationships modeled between the outcome variable and predictor variables are not consistent (either due to non-stationarity or heteroskedasticity). Hence, the local models were used as they assume that there is spatial heterogeneity between the independent and dependent variables. Moreover, residuals were not spatially clustered in locations (Global Moran’s I index value: 0.030339, *p*-value: 0.460773, z-score: 0.737574).

**Table 3:** Ordinary least square (OLS) model parameter estimate of not receiving Vit- A among children aged 6–35 months in Ethiopia, EMDHS 2019.

Variable	Coefficient	StdError	t-Statistic	Probability	Robust_SE	Robust_t	Robust_Pr	VIF
Intercept	−0.018401	0.202904	−0.090689	0.927788	0.143779	−0.127982	0.898239	--------
Maternal age 15–24	0.130182	0.091720	1.419345	0.156866	0.168630	0.771997	0.440725	5.681984
No maternal education	0.227024	0.054368	4.175666	0.000044*	0.100337	2.262599	0.024374*	4.727874
Protestant	0.211838	0.029352	7.217171	0.000000*	0.057843	3.662272	0.000307*	1.774631
Rural resident	0.014296	0.034660	0.412450	0.680322	0.070104	0.203918	0.838556	4.521284
Female household head	0.184009	0.088174	2.086879	0.037748*	0.187570	0.981016	0.327378	1.630764
Poorer/poorest household	0.255335	0.052552	4.858704	0.000003*	0.111821	2.283437	0.023102*	5.053190
Parity one	0.252603	0.057368	4.403171	0.000018*	0.120026	2.104575	0.036164*	4.361378
Female child sex	0.466447	0.064059	7.281483	0.000000*	0.103659	4.499829	0.000012*	5.849981
Child age 6–23 months	−0.366018	0.068660	−5.330855	0.000000*	0.139379	−2.626064	0.009083*	4.859130

OLS model diagnostics		
Diagnostic criteria	Values	Pr-
Akaike’s Information Criterion (AICc)	1505.935552	–
Multiple R-Squared	0.857648	–
Adjusted R-Squared	0.853305	–
Joint F-Statistic	197.480115	0.000000*
Joint Wald Statistic	599.063261	0.000000*
Koenker (BP) Statistic	95.176034	0.000000*
Jarque-Bera Statistic	197.480115	0.000000*

As each explanatory variable had a VIF of less than 7.5, we did not observe evidence of multicollinearity. All the coefficients of predictor variables have either negative or positive values, and some of them were statistically significant. Since the Koenker (BP) Statistic was statistically significant (p-value: 0.001), we relied on the Robust Probabilities (Robust_Pr) to determine coefficient significance.

The OLS regression analysis result showed that being from an uneducated mother, a protestant follower, being from a female household head, being from a poor/poorer household wealth status, being the first child, and a child aged 6–23 months were the significantly associated predictors for not receiving Vit-A supplement (Table 3). Child aged 6–23 months was negatively associated, but the remaining variables were positively related to not receiving Vit-A supplements.

### Geographically weighted regression analysis (GWR) and the multiscale extension (MGWR)

From the OLS regression analysis result, the Koenker (BP) and Jarque-Bera Statistic were statistically significant, indicating non-stationary among variable relationships and non-normal distribution of residuals, respectively. Thus, the OLS model prediction was biased. Therefore, we applied a spatially nonstationary local modeling approach namely GWR and MGWR, using the same sets of explanatory variables utilized in the OLS model. Both the local models explore the local spatial variation in relation to the outcome variable.

Since the GWR model can estimate the relationship between variables having different coefficients for each geographic unit, it is possible to map where the relations are weak and strong, significant and insignificant. Nevertheless, the GWR considers a single bandwidth for all parameters and operates at that single spatial scale. To overcome this limitation, a Multiscale Geographically Weighted Regression (MGWR) model was used. The model allows the geographic variation of the relationship between dependent and explanatory variables, considering multiple bandwidths. Model diagnostic information is presented in Table 4.

**Table 4:** Diagnostic information for the GMR and MGWR models for non-receipt of Vit-A supplement among children 6–35 months in Ethiopia, EMDHS 2019.

Explanatory variables	Maternal age 15–24, no maternal education, protestant follower, rural resident, female household head, poor/poorer wealth status, parity one, female, child age
GWR	
Neighbors	135
Residual Squares	1077.264855
Effective Number	51.44941
Sigma	2.061242
AICc	1349.417146
R-Squared	0.920393
Adjusted R-Squared	0.920393
MGWR	
Residual sum of squares	17.888
Sigma estimate	0.265
AICc	124.453
R-Squared	0.941
Adjusted R-Squared	0.930

The summary statistics of estimated coefficients of the local terms (MGWR model), as well as the optimal bandwidth for each predictor, are described in Table 5.

**Table 5:** Summary statistics for MGWR parameter estimates.

Variable	Mean	STD	Min	Median	Max	Bandwidth
Intercept	−0.050	0.166	−0.320	−0.109	0.585	44.000
Maternal age 15–24	0.107	0.108	−0.058	0.177	0.225	127.000
No maternal education	0.385	0.003	0.380	0.384	0.391	304.000
Protestant	0.137	0.004	0.127	0.137	0.141	304.000
Rural	0.015	0.002	0.010	0.016	0.019	304.000
Female HH head	−0.077	0.064	−0.171	−0.092	0.059	142.000
Poorer/poorest household	0.103	0.161	−0.254	0.111	0.434	46.000
Parity one	0.303	0.124	0.070	0.348	0.553	75.000
Female child sex	0.251	0.004	0.245	0.250	0.259	304.000
Child_Ag_1	−0.326	0.058	−0.388	−0.357	−0.177	145.000

### Model performance comparison

We compared the performance of the three models using Akaike’s Information Criterion (AICc) and Adjusted R-squared to determine the best model fit. Accordingly, both the GWR and MGWR models demonstrate better performance than the global model. The Adjusted R^2^ improved from 85.33% in OLS to 92.04% in GWR analysis and 93.0% in MGWR, indicating that the local models better explained the spatial variations of not receiving Vit-A supplement among children 6–35 months in Ethiopia. Furthermore, the AICc value of 1505.93 in OLS was reduced to 1349.42 in GWR, which also indicates a better model fit (Table 6). As the model diagnostics indicate, the MGWR model performed better than the OLS and GWR models and was selected as the best fit. A detailed explanation is depicted in Table 6.

**Table 6:** Model fitness comparison between the global and local models.

Model comparison parameters	OLS	GWR	MGWR
Akaike’s Information Criterion (AICc)	1505.935552	1349.417146	294.173
Multiple R-Squared	0.857648	0.920393	0.941
Adjusted R-Squared	0.853305	0.920393	0.930

### Mapping the parameter coefficients

Concerning the spatial predictors of not receiving Vit-A supplement among children 6–35 months, both GWR and MGWR models’ covariate estimates were mapped to show their local effect in each neighborhood and their geographical distribution. Accordingly, being from an uneducated mother, having poor wealth status, being the first child, and child aged 6–23 months were strong and weak predictors of not receiving Vit-A supplements among children aged 6–23 months. The coefficient estimates were mapped from bright red (indicating strong association) to green (representing weak association).

**Figure 7:**
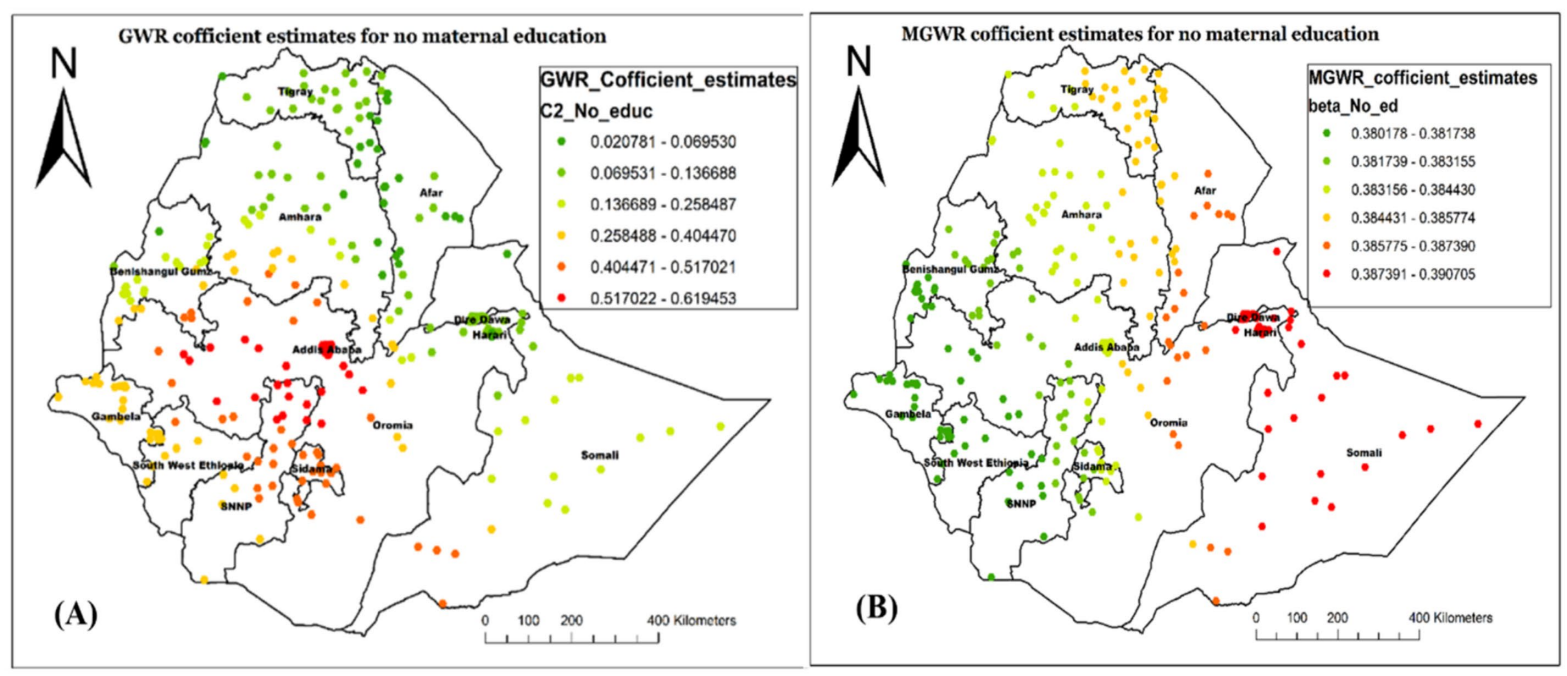
Local coefficient estimates of no maternal education for not receiving Vit-A supplement among children 6–35 months in Ethiopia, EMDHS 2019.

The coefficient estimates of being from an uneducated mother spatially vary across the study area, indicating both weak and strong associations. In both the local models, the variable showed a positive association in describing not having Vit-A supplementation (Figures 7A,B) indicating that being from an uneducated mother increases the likelihood of not receiving Vit-A supplement. As illustrated in Figure 7, the variable has the highest coefficient value in Somalia, Diredawa, and Harari regions, implying that it is a strong predictor for not receiving Vit-A supplements in those specified areas. On the contrary, the lowest coefficient value of no maternal education is located in Northeast Oromia, Southwest Ethiopia, and most of Gambela as well as Benshangul Gumz.

**Figure 8:**
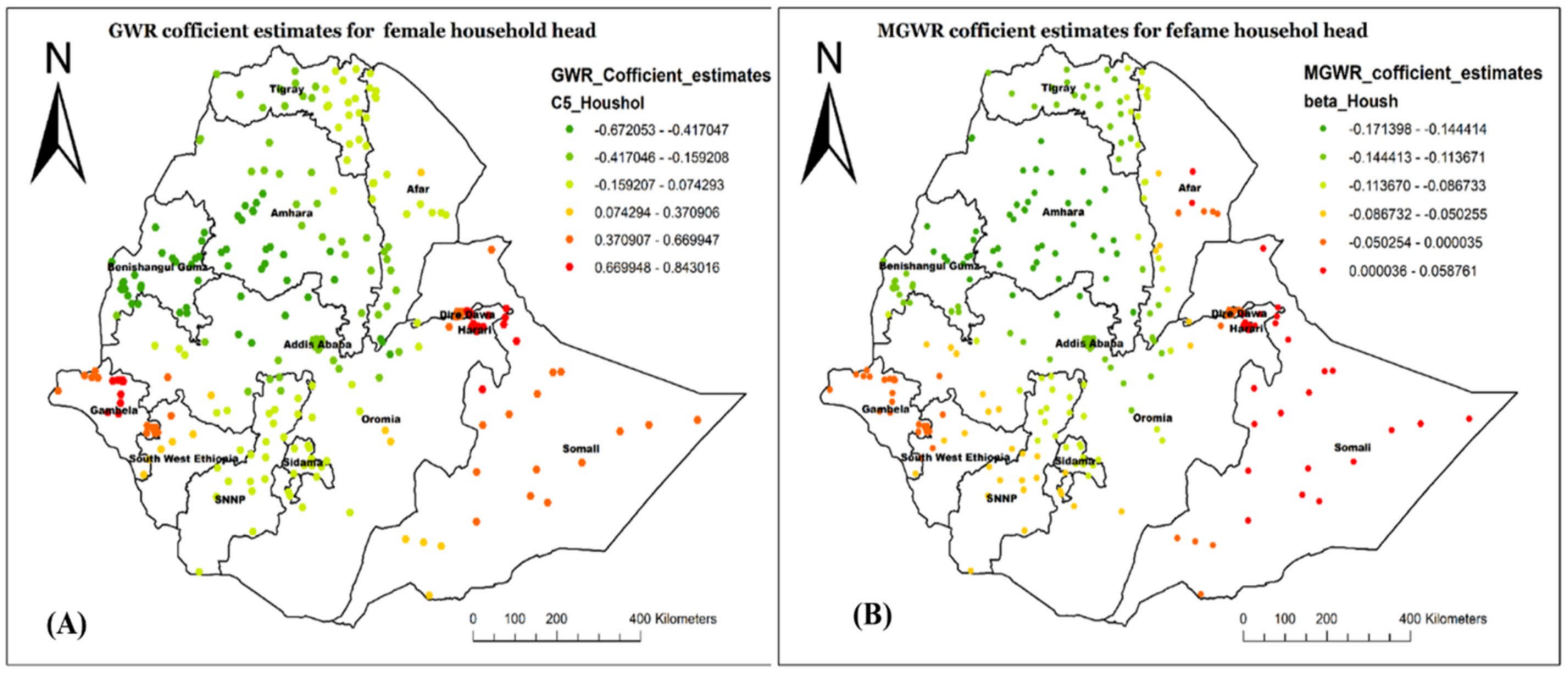
Local coefficient estimates of female household heads for not receiving Vit-A supplement among children 6–35 months in Ethiopia, EMDHS 2019.

Figure 8 demonstrated a similar geographic distribution estimate of GWR (A) and MGWR (B) for the child being from a female household head. The coefficient ranged from −0.171 to 0.059 in the MGWR model result, indicating that it tends to have both negative and positive associations with the outcome variable (Figure 8). Overall, the variable has a negative association with not receiving Vit-A supplement, with an average value of −0.077. A higher positive association was observed in Western Afar, Harari, and most of the Somalia region. There were also some positive estimates in Diredawa and Gambela Region.

**Figure 9:**
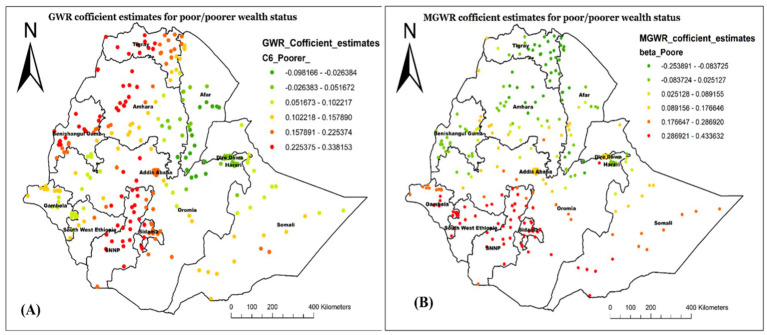
Local coefficient estimates of poor/poorer household wealth status for not receiving Vit-A supplement among children 6–35 months in Ethiopia, EMDHS 2019.

Figure 9 shows the regression coefficient estimate of being from a poor/poorer household, which describes the spatial variations in the influence of this predictor on not having Vit-A supplement in different Regions. In the MGWR model (Figure 9B), the coefficient estimate ranged from −0.254 to 0.434 with an average value of 0.103 (Table 5). The red-colored cluster points (located central to Northwest Gambela, Southwest Oromia, Southwest Somali, most of SNNP, and Sidama) imply an area where there is a strong positive relationship between poor household wealth status and not receiving Vit-A supplement among children aged 6–35 months. This finding indicates that children who were from poor households were more likely not to receive Vit-A supplements in those mentioned areas.

**Figure 10:**
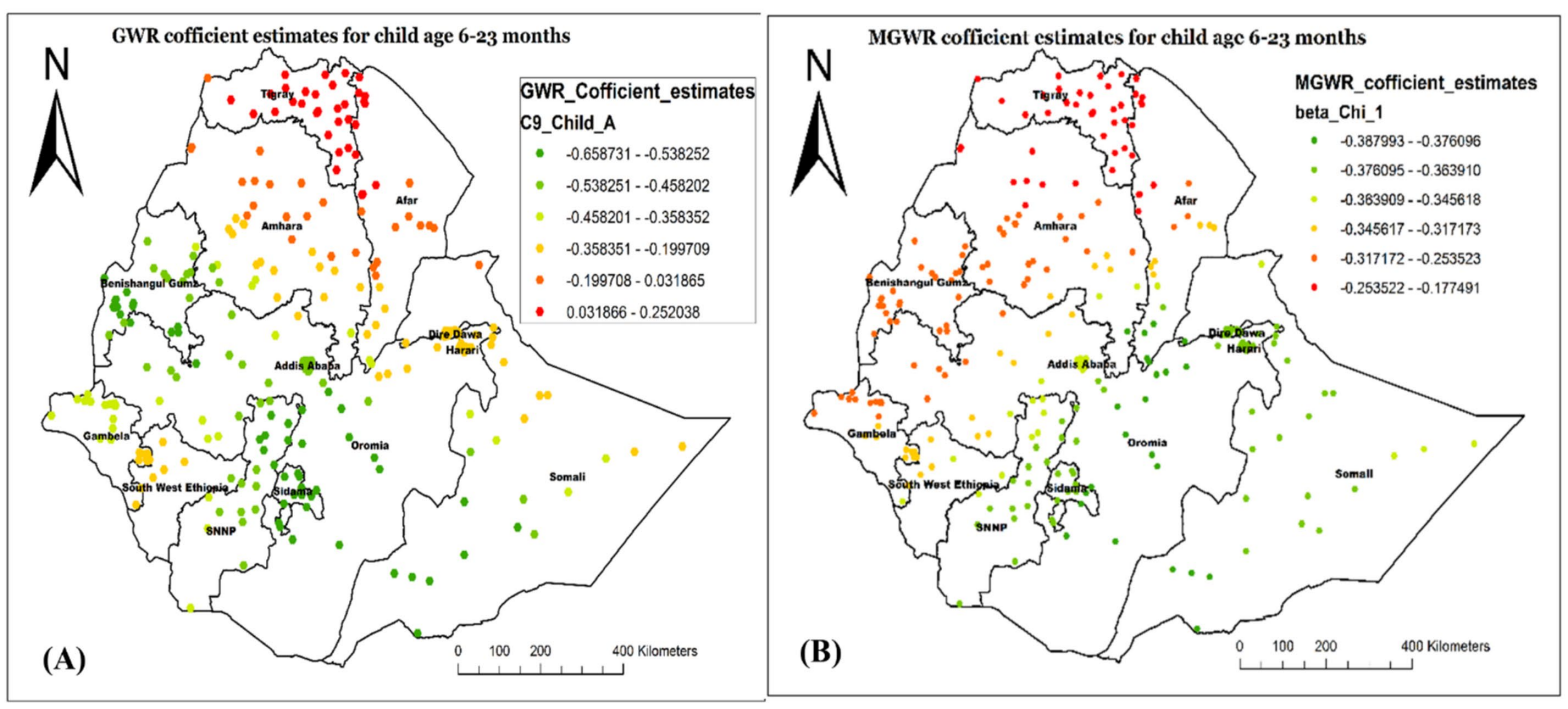
Local coefficient estimates of children aged 6–23 months for not receiving Vit-A supplement among children 6–35 months in Ethiopia, EMDHS 2019.

Figure 10 also proved the GMR- (Figure 10A) and MGWR- (Figure 10B) coefficient estimate for children aged 6–23 months. As the MGWR model result revealed, the parameter estimates for this variable tend to have a range of negative spatial structures, indicating that children aged 6–35 months tend to have a lower chance of not receiving Vit-A supplements across the country. Specifically, the higher negative effect of children aged 6–23 months age on not receiving Vit-A supplements was observed in Northwest Afar, and most parts of Amhara, Tigray, and Afar regions. The GWR parameter estimate also showed spatial heterogeneity. The model showed that children aged 6–35 months tend to have a lower chance of not receiving Vit-A supplements in Southwest Somali, Central Oromia, Addis Ababa, most of Sidama, and SNNP.

**Figure 11:**
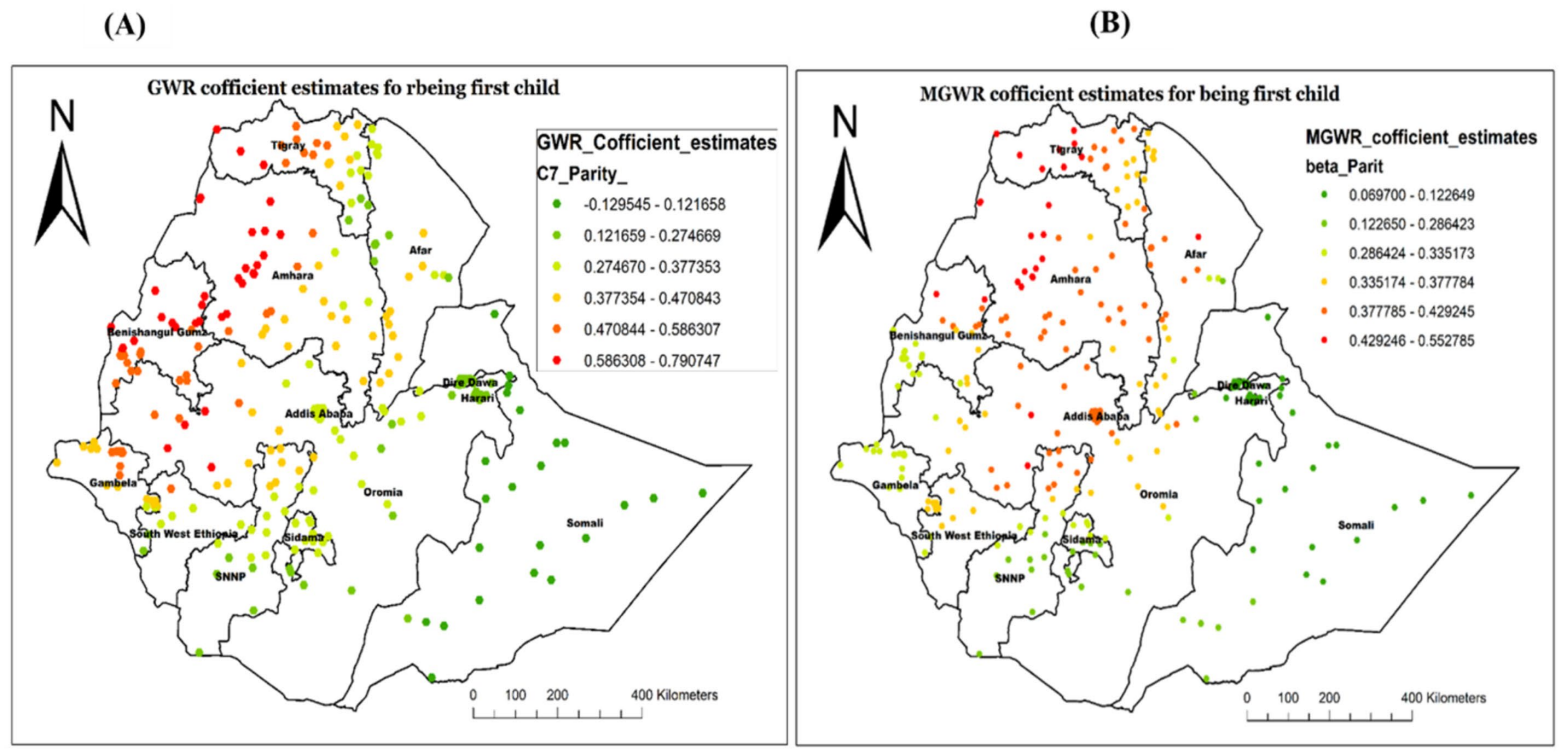
Local coefficient estimates of being a first child for not receiving Vit-A supplement among children 6–35 months in Ethiopia, EMDHS 2019.

The analysis result of this study also revealed the GWR and MGWR coefficient estimates of being the first child, with the range of high GWR positive coefficient values found in the Northwest of the study area, specifically in Tigray, Northwest Amhara, and Benishangul Gumz. This implied that being the first child increases the likelihood of not receiving Vit-A in those specified areas. Conversely, the coefficient estimates were found to be smaller in most of Somali, Diredawa, Hareri and some parts of Afar, indicating a smaller spatial relationship (Figure 11).

## Discussion

In many developing countries, including Ethiopia, inadequate coverage of Vit-A supplementation continues to be a serious public health concern. Although Vit-A has been shown to reduce mortality and morbidity of the child, many places in Ethiopia still lack appropriate coverage because of several factors. Identifying and reducing the preventable underlying determinants is there for an essential plan of action to improve the Vit-A supplementation rate. Hence, this study aimed to explore the spatial distribution and determinants of non-receipt of Vit-A supplement among children aged 6–35 months using geospatial models.

In this study, the all over prevalence of not having Vit- A supplements among children 6–35 months in Ethiopia was found to be 53.2% (95% CI: 51.0, 55.1%). This prevalence varied across the country’s administrative Regions, ranging from 37.7% in the Amhara region to 77.9% in the Somali Region. This variation was supported by the statistically significant Global Moran’s statistics, which confirmed a non-random spatial distribution. This implies that the proportion of not receiving Vit-A supplement among children 6–35 months was significantly varied from one place to the other place, throughout the country.

The hotspot analysis identified the high and low-risk areas of not having Vit -A supplements among children 6–35 months. The high prevalence of not receiving Vit-A supplements was identified in Oromia, Sidama, and SNNP. Whereas the low prevalence was observed in Dire Dawa, Harari, Banshangil Gumz, and Gambela Regions. Similarly, the ordinary kriging interpolation prediction of the unsampled area also showed that the highest rates of not receiving Vit-A supplements were located in central Oromia and some parts of Sidama Regions. This observed spatial variation may be due to a disparity in health service access and nutritional interventions as well as cultural and geographic challenges. In regions like part of Oromia and Somali, traditional beliefs, and mobility of pastoralist communities can limit access to healthcare, particularly for children (25). The geographical and environmental challenges in these regions also influence basic health service delivery including Vit – A supplementation campaigns. It has been reported that Geographical features in regions like SNNP and Sidama, with challenging topographies, health service distribution is affected, making them less accessible, resulting in lower coverage rates for remote communities (26, 27). Moreover, a difference in parental sociodemographic factors and lack of awareness among caregivers about Vit – A supplements play a significant role in the disparity (12, 28, 29).

In our spatial regression analysis, the MGWR model overcame the limitations of OLS as well as GWR and resulted in a better-fitted model in this study. Accordingly, being from an uneducated mother, being from a poor wealth status of household, being the first child, and being a young child age were spatially varied, strong and weak predictors of not receiving Vit-A supplements among children 6–35 months.

The MGWR analysis revealed that being from an uneducated mother was positively associated with never receiving Vit- A supplement (both strong and weak association). A strong association was observed in Dire Dawa, Harari, and the Somali Region. This is potentially due to mothers with lower educational levels may not be aware of the importance of Vit A, are less likely to engage with healthcare services, including nutritional supplements, and may not be well equipped to navigate health information (30, 31). Our finding that children from uneducated mothers were less likely to take vitamin A supplements is consistent with earlier studies, indicating that maternal education is a significant factor (29, 32).

Similarly, the MGWR model showed that being from a female-headed household was found to have a significant positive association with not having Vit -A supplement in Central Afar, Hareri and most of the Somali Region. This could be due to female-headed households facing cultural challenges, limiting access to healthcare services, as well as nutritional practices (33, 34). Traditional gender roles, social stigma, and cultural norms may marginalize children’s health needs, affecting the prioritization of health interventions. They all result in lower coverage of essential health interventions, including vital nutrient supplementations (35, 36).

The findings of this study from the MGWR analysis also showed that, on average, being from a poor/poorest household positively affects the likelihood of not having Vit -A supplement. The highest association was identified in the Southwest of Oromia, Southwest Somali, Central to Northwest Gambela, most of SNNP and Sidama regions. This is consistent with previous studies highlighting the influence of socioeconomic status on healthcare service accessibility, including nutritional interventions (11, 37, 38). The lower uptake of Vit-A supplementation among children 6–35 months from poor households, perhaps in part because of low socioeconomic level, affects health-seeking behaviors, including nutritional interventions (39). Better household wealth may improve the intake of Vit-A supplements by improving health information accessibility and reducing economic barriers (11, 40). It has also been reported that socioeconomic inequality significantly affects maternal health service utilization, with poor families showing higher vitamin A deficiency in their children (41, 42). Additionally, according to recent reports, regions like some parts of Oromia, Sidama, and the western part of SNNP face significant disparities in healthcare provision, leading to lower rates of Vitamin A supplementation among children (43).

Our study also highlighted that being a first child had a predominantly positive influence on not accessing Vit-A supplementation among the study participants. The highest association was identified in Western Tigray, Northwest Amhara, Northern Benishangul Gumz, and some areas in Western Oromia. From previous studies, it has been reported that first-born children are less likely to receive nutritional interventions, including Vit-A supplementation (29, 44). This might be due to the presence of some factors, including a lack of experience and awareness about the importance of essential nutrient supplementation for first-time parents. Additionally, first-time parents may be less familiar with healthcare systems, leading to missed opportunities for Vit- A supplementation. Studies showed that parental awareness is crucial for ensuring child nutritional interventions, while younger siblings receive more attention (45, 46).

### Recommendations

The findings of this study highlight the need for targeted interventions to reduce the spatial disparities in Vit-A supplementation among children aged 6–35 months in Ethiopia. Public health programs should prioritize high-prevalence regions, particularly Sidama, SNNPR, and parts of Oromia, to improve coverage. Efforts to increase maternal education and awareness about child nutrition are crucial, especially for mothers with lower educational levels. Special attention should also be given to vulnerable households, including female-headed and poorer households, as well as younger children (6–23 months) and first-born children, who are at higher risk of missing supplementation. Policymakers should use these spatial and sociodemographic insights to design geographically targeted and socially inclusive strategies to enhance Vitamin A supplementation nationwide.

### Strengths and limitations of the study

The study was based on nationally and regionally representative data enhancing the reliability and generalizability of the findings. Furthermore, the use of spatial analysis including spatial local modeling, which helps to identify spatially varying relationships between predictors was another strength. However, this study has some limitations that must be considered during interpretation. Since this study is based on secondary data analysis, some determinants were not included; hence, we failed to adjust their effect. The study utilized cross-sectional survey data, limiting the causal relationships between identified predictors and the outcome variable. In addition, since the data is based on self-reporting, social desirability bias and recall bias might be present.

## Conclusion

This study provided the spatial disparities and determinants of not having Vit- A supplements among children aged 6–35 months using the 2019 EMDHS data. The finding revealed a significant geographical variation in the non-receipt of Vit-A supplement, with some areas like Sidama, SNNPR, and some parts of Oromia exhibiting a higher prevalence of not receiving Vit-A supplementation. In the spatial regression analysis, some socioeconomic factors, such as being from an uneducated mother, having poor wealth status, being a first child, and having a child aged 6–23 months, were strong and weak predictors (at the local level) in different regions of Ethiopia. These factors varied significantly across different regions, emphasizing the importance of context-specific strategies to improve Vit-A supplementation. Therefore, targeting public health strategies in the identified hotspot areas has to be implemented, with consideration of the identified determinants, to improve Vit-A supplementation coverage.

## Data Availability

The datasets presented in this study can be found in online repositories. The names of the repository/repositories and accession number(s) can be found at: https://dhsprogram.com/data/dataset.
