# Supplementary material for: Spatial distribution and determinants of Vitamin A supplementation non-receipt among children aged 6–35 months in Ethiopia: a multiscale geographically weighted regression analysis
Source: Front Public Health. 2025 Oct 31;13:1483588. doi: 10.3389/fpubh.2025.1483588 (PMC12617433; doi:10.3389/fpubh.2025.1483588)
Supplement: Supplementary file 1 [file Supplementary_file_1.docx]

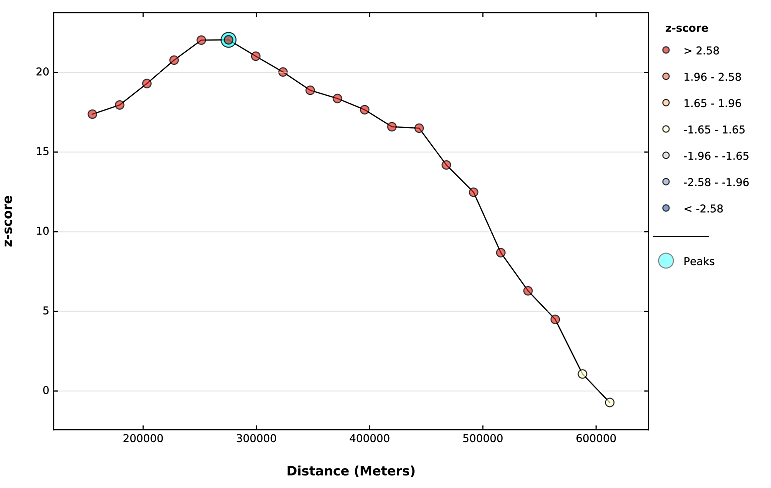


Figure one. Incremental autocorrelation of non-receipt of Vit-A supplement among children aged 6-35 months in Ethiopia, EMDHS 2019.
