# Supplementary material for: Spatial distribution and determinants of Vitamin A supplementation non-receipt among children aged 6–35 months in Ethiopia: a multiscale geographically weighted regression analysis
Source: Front Public Health. 2025 Oct 31;13:1483588. doi: 10.3389/fpubh.2025.1483588 (PMC12617433; doi:10.3389/fpubh.2025.1483588)
Supplement: Supplementary file 2 [file Supplementary_file_2.docx]

Table 1. Spatial SaTscan analysis result of not receiving Vit-A supplementation among children aged 6-35 months in Ethiopia, EMDHS 2019.

| **Clusters** | **Enumeration area** | **Co-coordinate/radius** | **Population** | **Cases (%)** | **RR** | **LLR** | **P-value** |
| --- | --- | --- | --- | --- | --- | --- | --- |
| Primary clusters-44 | 190, 189, 191, 197, 178, 204, 198, 184, 115, 180, 199, 182, 172, 179, 188, 173, 187, 185, 177, 181, 186, 196, 203, 113, 183, 91, 89, 116, 117, 192, 95, 176, 205, 96, 202, 174, 171, 195, 97, 175, 112, 201, 114, 194 | (6.745024 N, 37.662745 E) / 241.91 km | 896 | 610(68.1) | 1.50 | 61.636037 | <0.001 |
| Secondary clusters -22 | 137, 138, 123, 135, 142, 136, 145, 134, 140, 131, 141, 122, 132, 133, 124, 125, 143, 144, 129, 111, 139, 121 | (5.856584 N, 43.726016 E) / 402.89 km | 160 | 125(78.1) | 1.51 | 22.525411 | <0.001 |
